# Supplementary material for: Atovaquone: An Inhibitor of Oxidative Phosphorylation as Studied in Gynecologic Cancers
Source: Cancers (Basel). 2022 May 5;14(9):2297. doi: 10.3390/cancers14092297 (PMC9102822; doi:10.3390/cancers14092297)
Supplement: Supplementary file 1 [file cancers-14-02297-s001.zip › cancers-1635970-supplementary.pdf]

Supplemental Figure S1

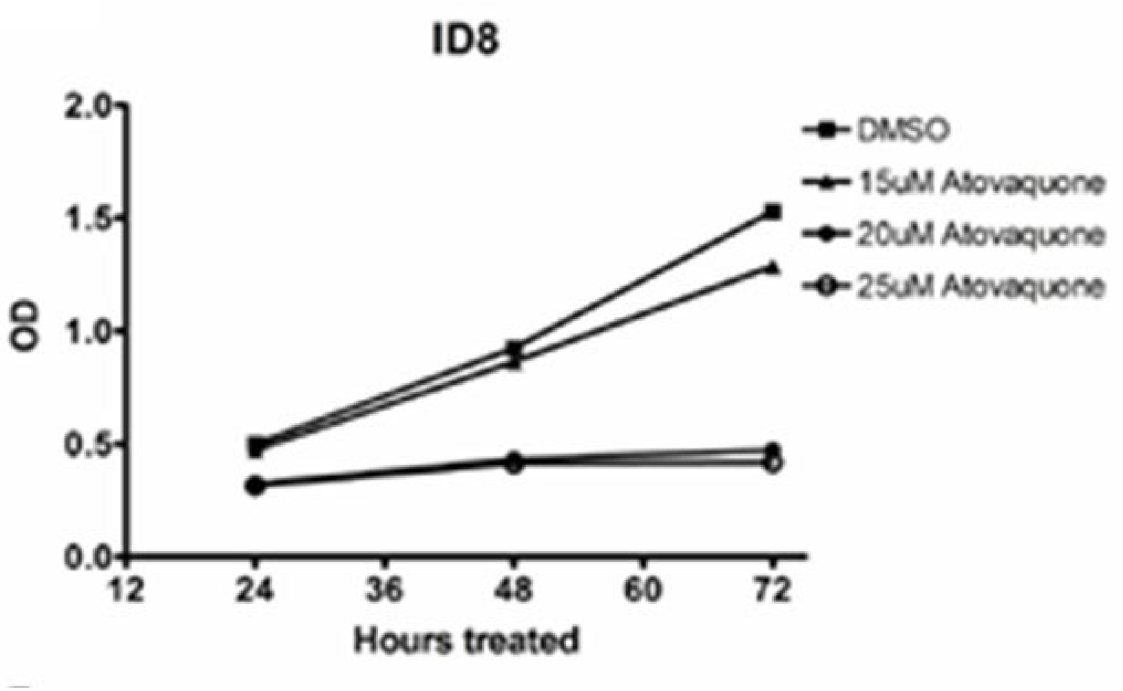

Supplemental Figure S2

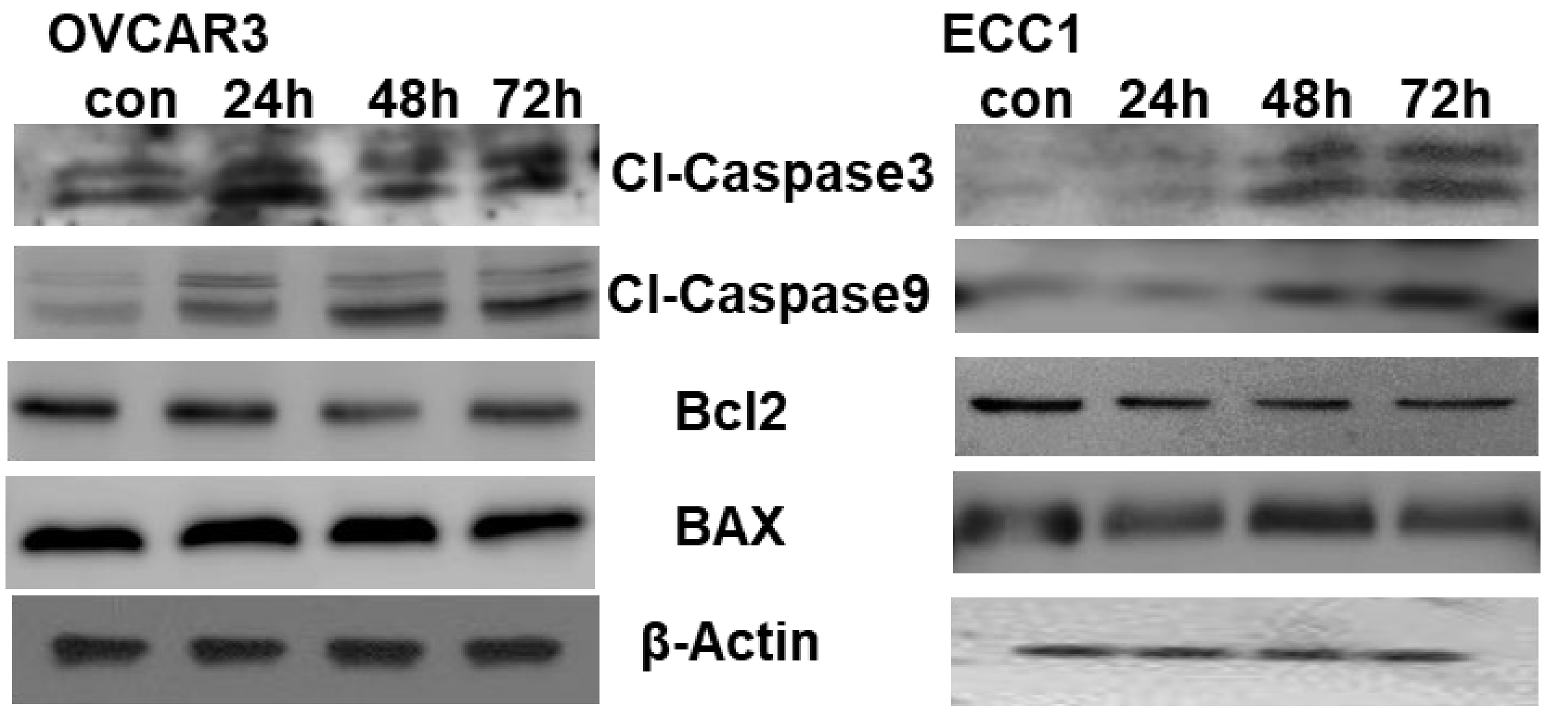

Supplemental Figure S2

OVCAR3 and ECC-1 cell lines were treated with atovaquone or DMSO, washed with ice-cold PBS and lysed in RIPA buffer containing protease inhibitors. Protein concentration was measured using BCA protein assay (ThermoFisher). Lysates equivalent to 25-30 g of electrophoresed, blotted to PVDF membranes and probed with primary and secondary antibodies. The protein bands were detected using chemiluminescence substrate.

Atovaquone increases apoptosis in OVCAR3 and ECC1 cells lines as indicated by an increase cleaved caspase 3 and 9 and a decrease in Bcl-2. An IC_{50} of atovaquone of 10 was used, which matches the human plasma concentration when this drug is used for malaria prophylaxis.

Supplemental Figure S3

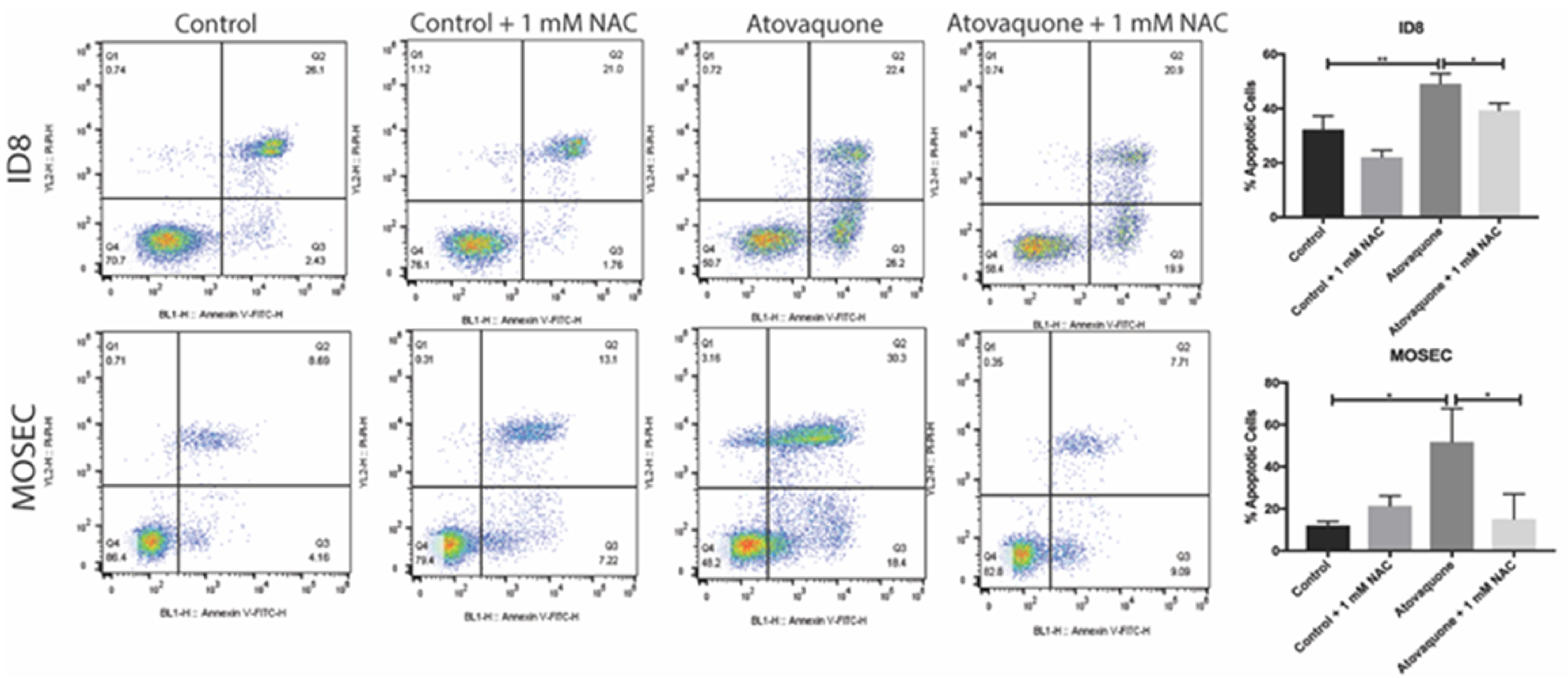

B

Supplemental Figure S3:

Atovaquone was found to rapidly increase intracellular oxygen radicals. Cells were pre-incubated for 30 minutes prior to treatment with atovaquone (10μM) with the oxygen radical scavenger, N-acetylcysteine (NAC). NAC attenuated the anti-proliferative and pro-apoptotic effect of atovaquone on ID8 and MOSEC cells, confirming a major mechanism by which atovaquone mediates its anti-cancer effects. Cells were stained with Annexin V-FITC and apoptosis was monitored by flow cytometry. */**, $p<0.05$apoptosis was monitored by flow cytometry (*/** $p <0.05$).

Supplemental Figure S4

ECC-1

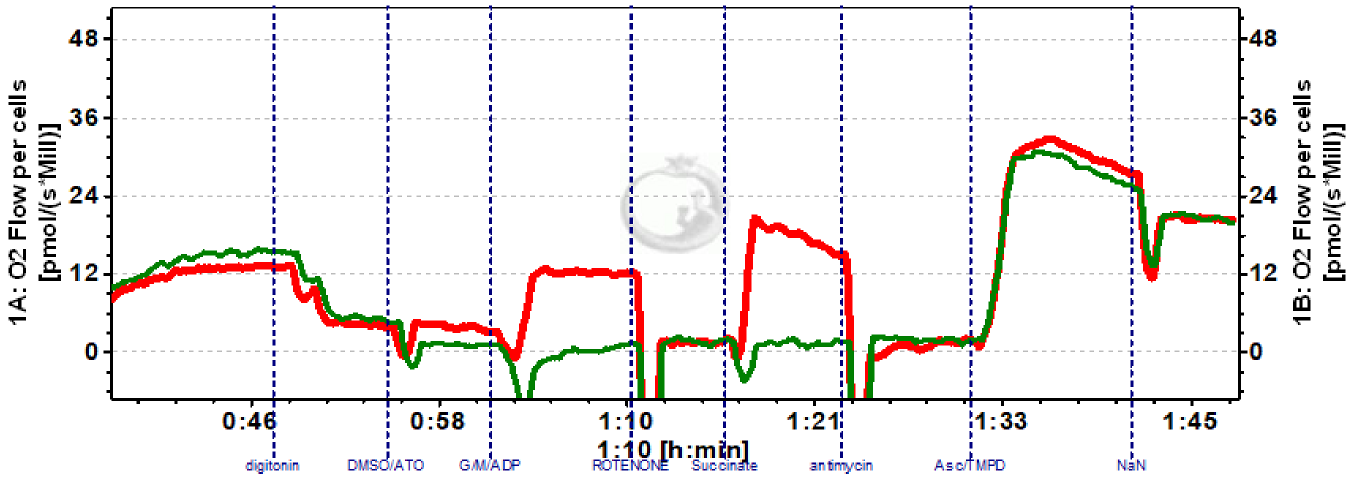

SKOV-3

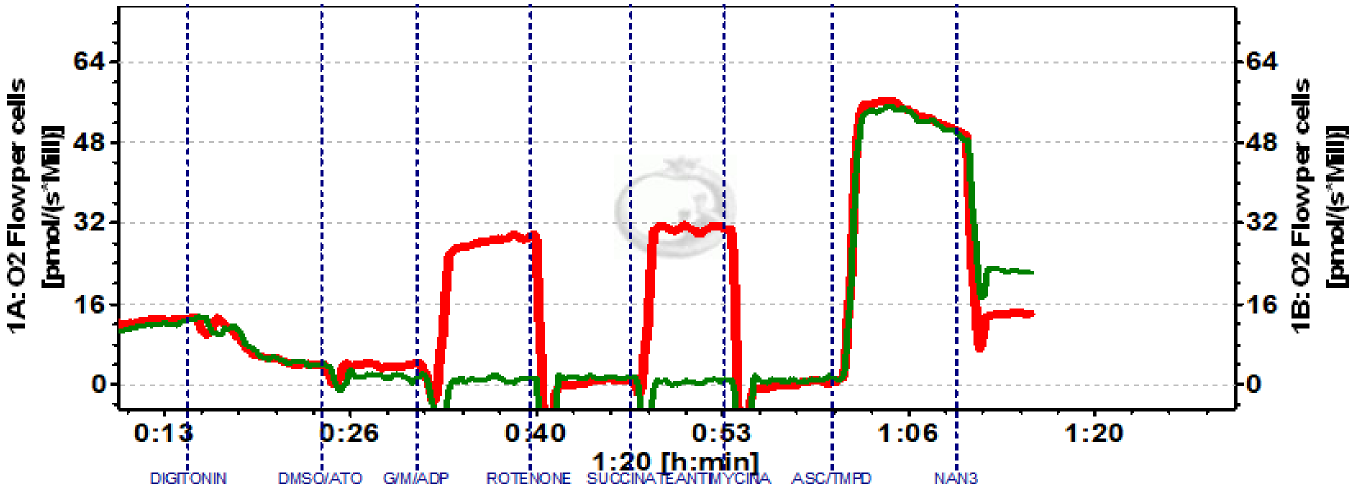

Supplemental Figure S4:

Inhibition of oxidative phosphorylation by Atovaquone (Ato) in ECC1 and SKOV3 cells was monitored on the Seahorse Xfe96 analyzer in triplicate. Data was normalized to the number of cells plated and cell viability was confirmed at the end of the experiment.

Supplemental Figure S5

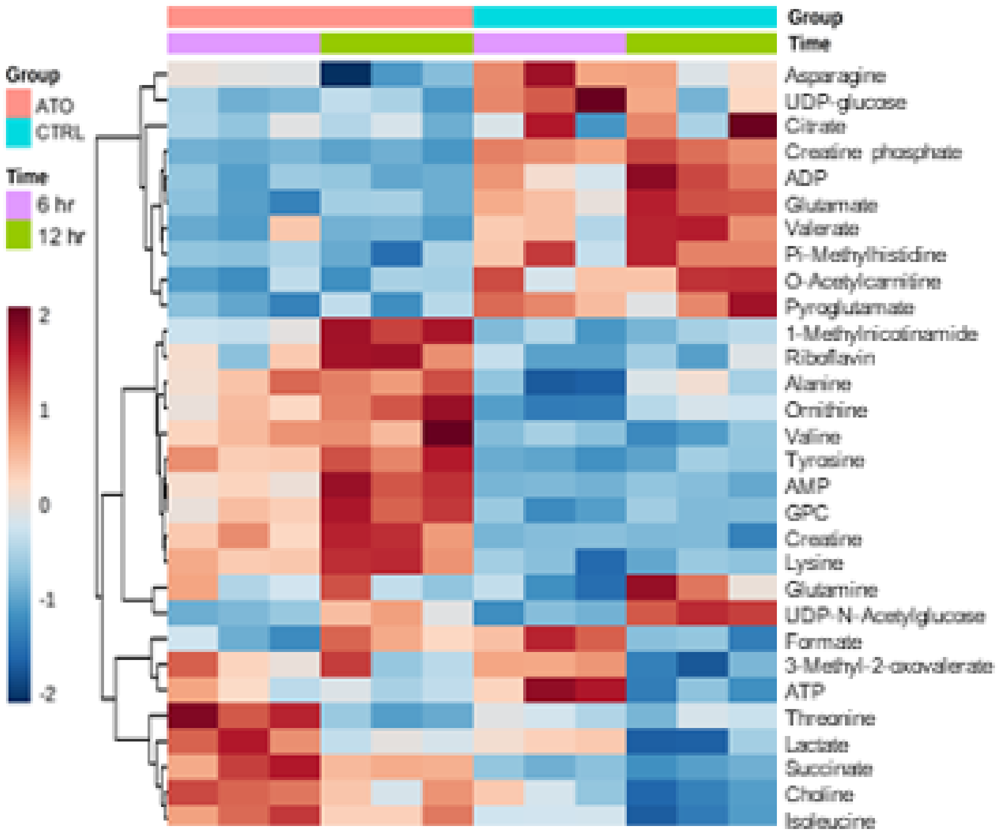

Supplemental Figure S5:
Heat map with hierarchical clustering of significantly altered metabolites for 6 and 12 hr of atovaquone (ATO) treatment and control (CTRL) samples (Pearson distance, complete linkages, ANOVA, FDR<0.05).

Supplemental Figure S6: Panel A

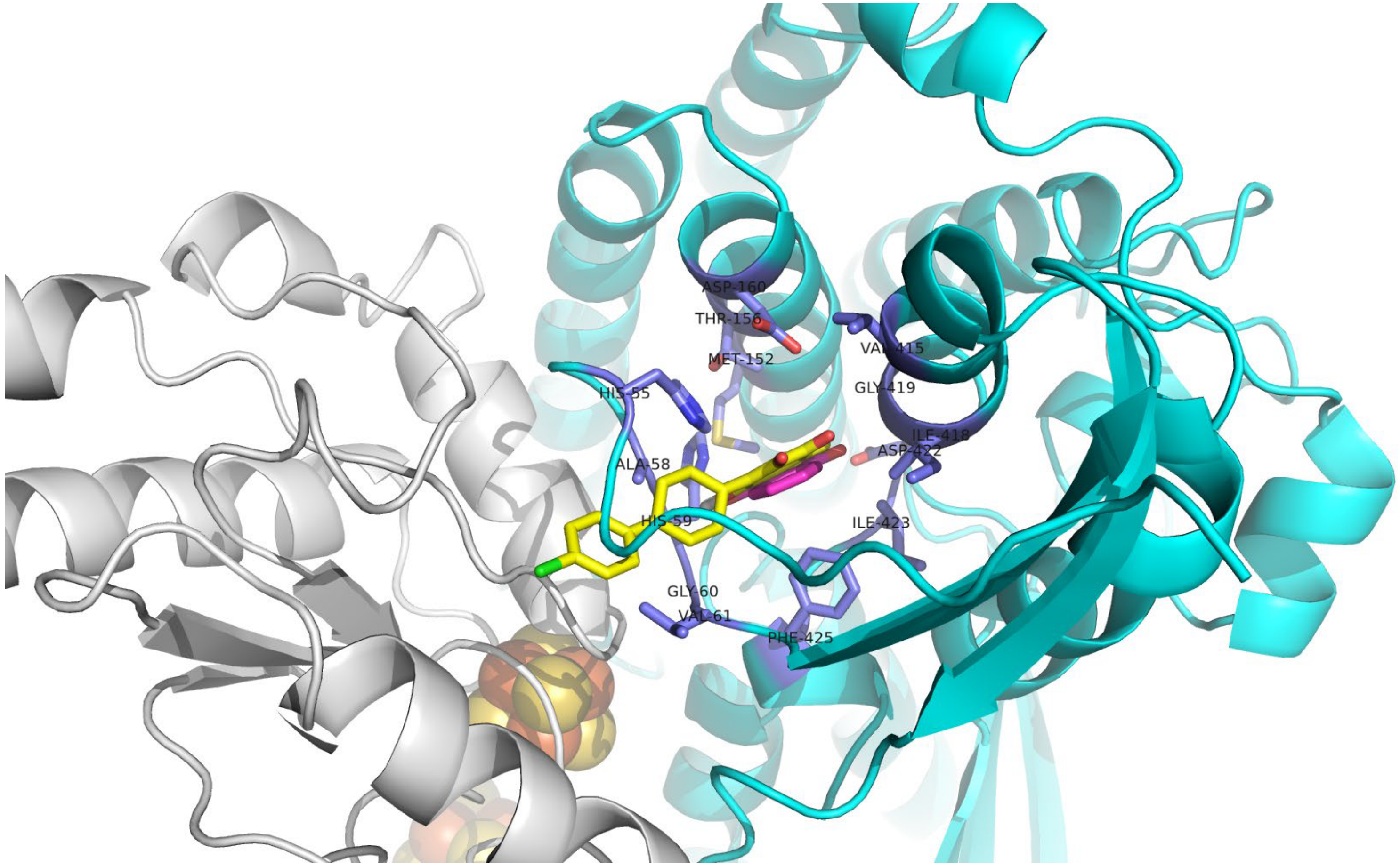

Supplemental Figure S6: Panel B

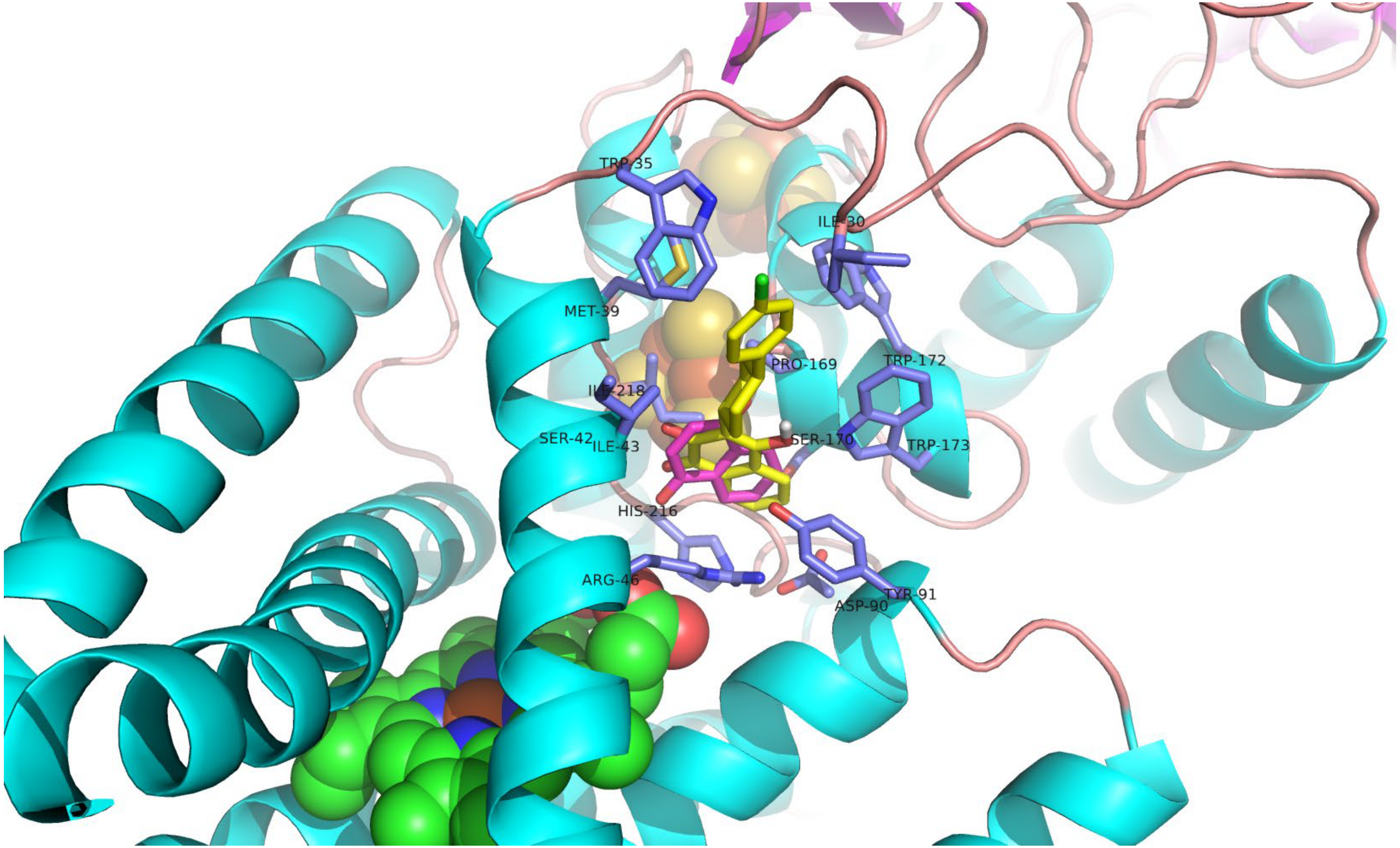

Supplemental Figure S6: Panel C

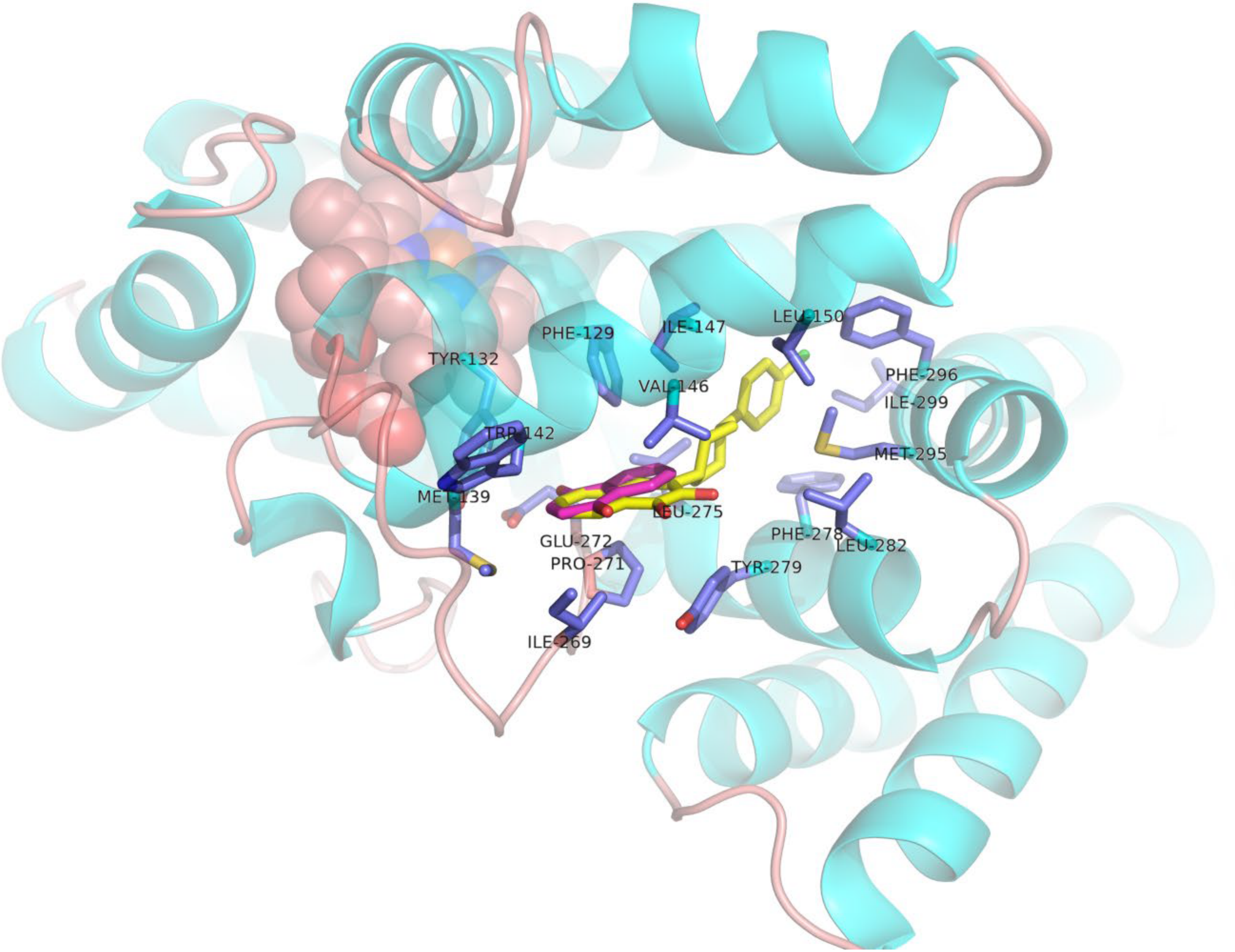

Supplemental Figure S6: Panel D

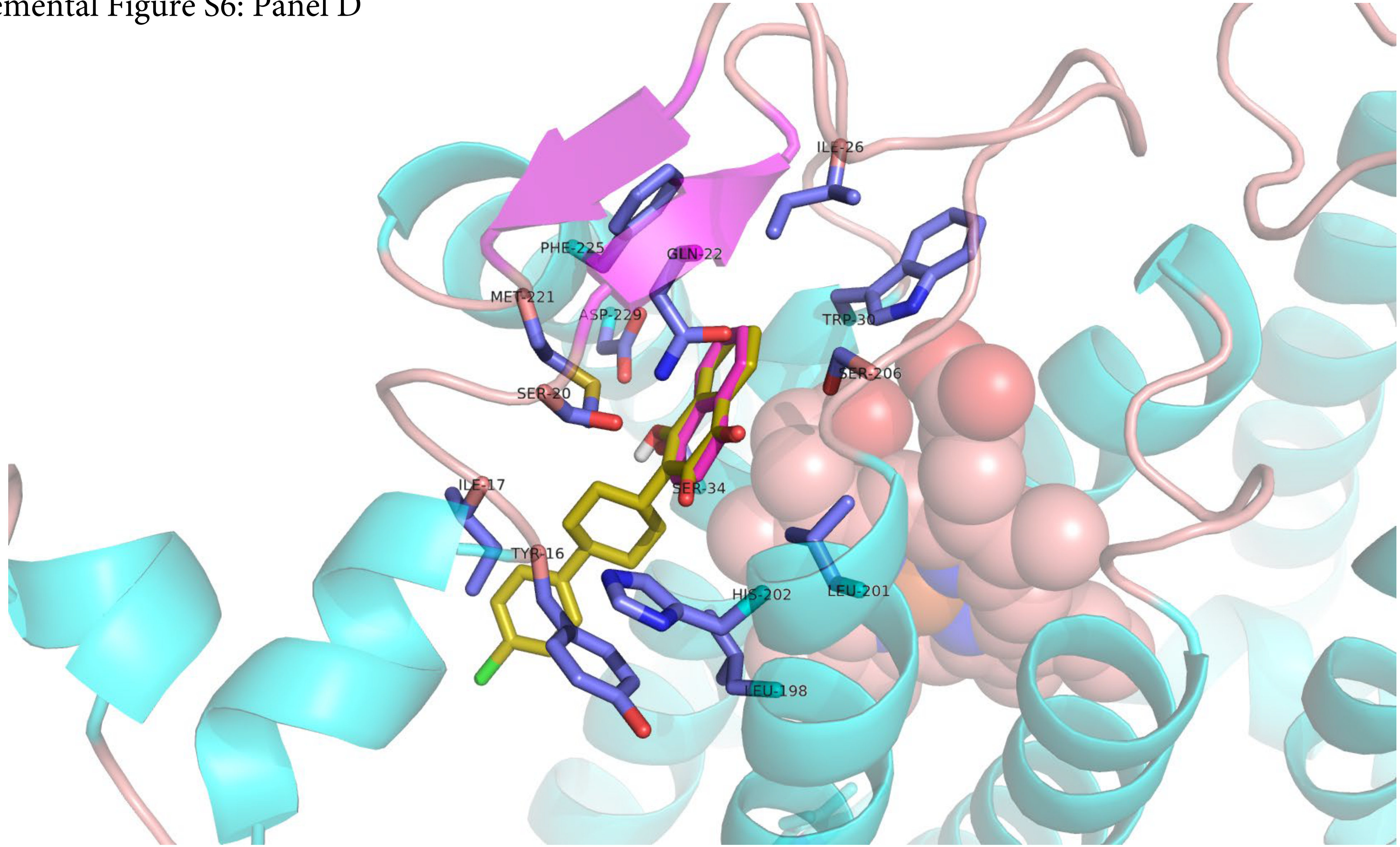

Supplemental Figure S6:
Docking of atovaquone in the Q0 and Qi sites of mitochondrial complexes III, (A) and (B), respectively. Docking of atovaquone to ubiquinone binding sites on mitochondrial complexes I and II are shown in (C) and (D), respectively.

Supplementary Figure S7

Figure 3B (Upper Panel)

ECC1

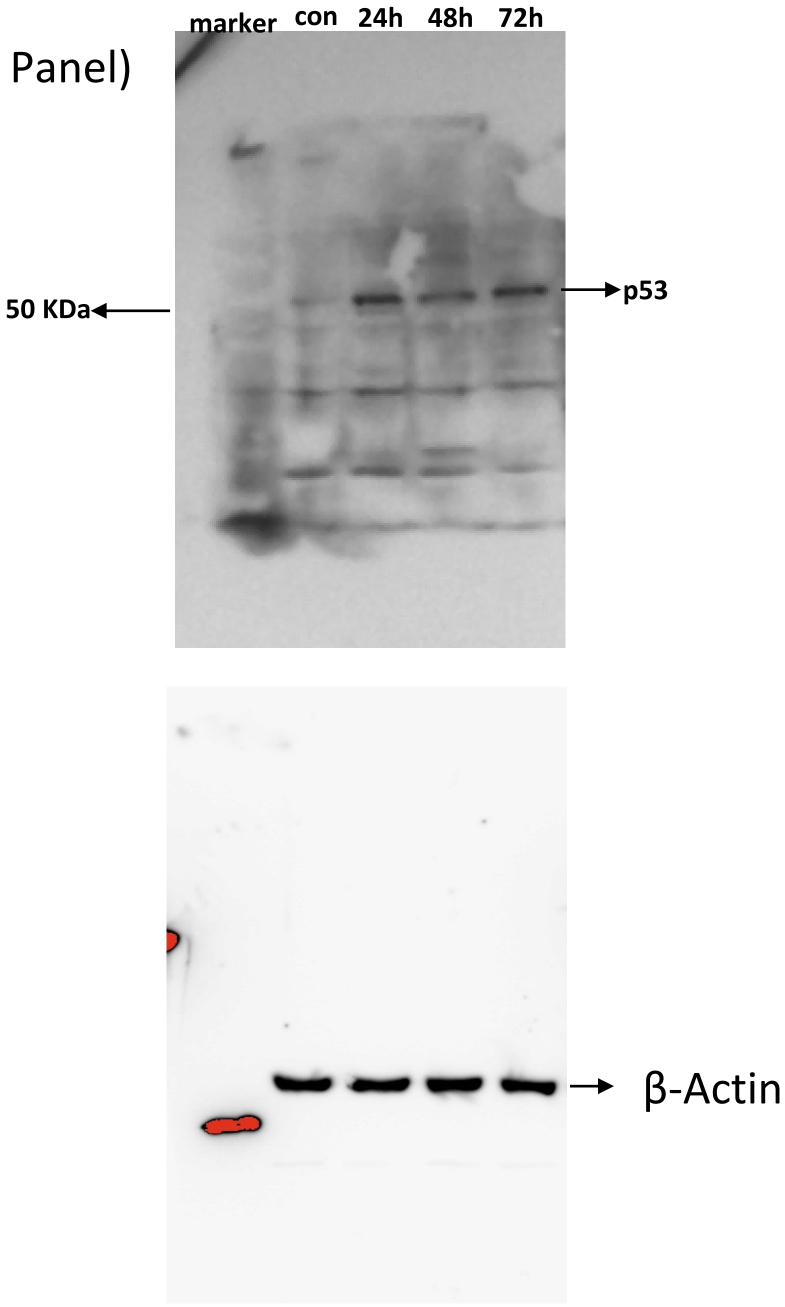

OVCAR3

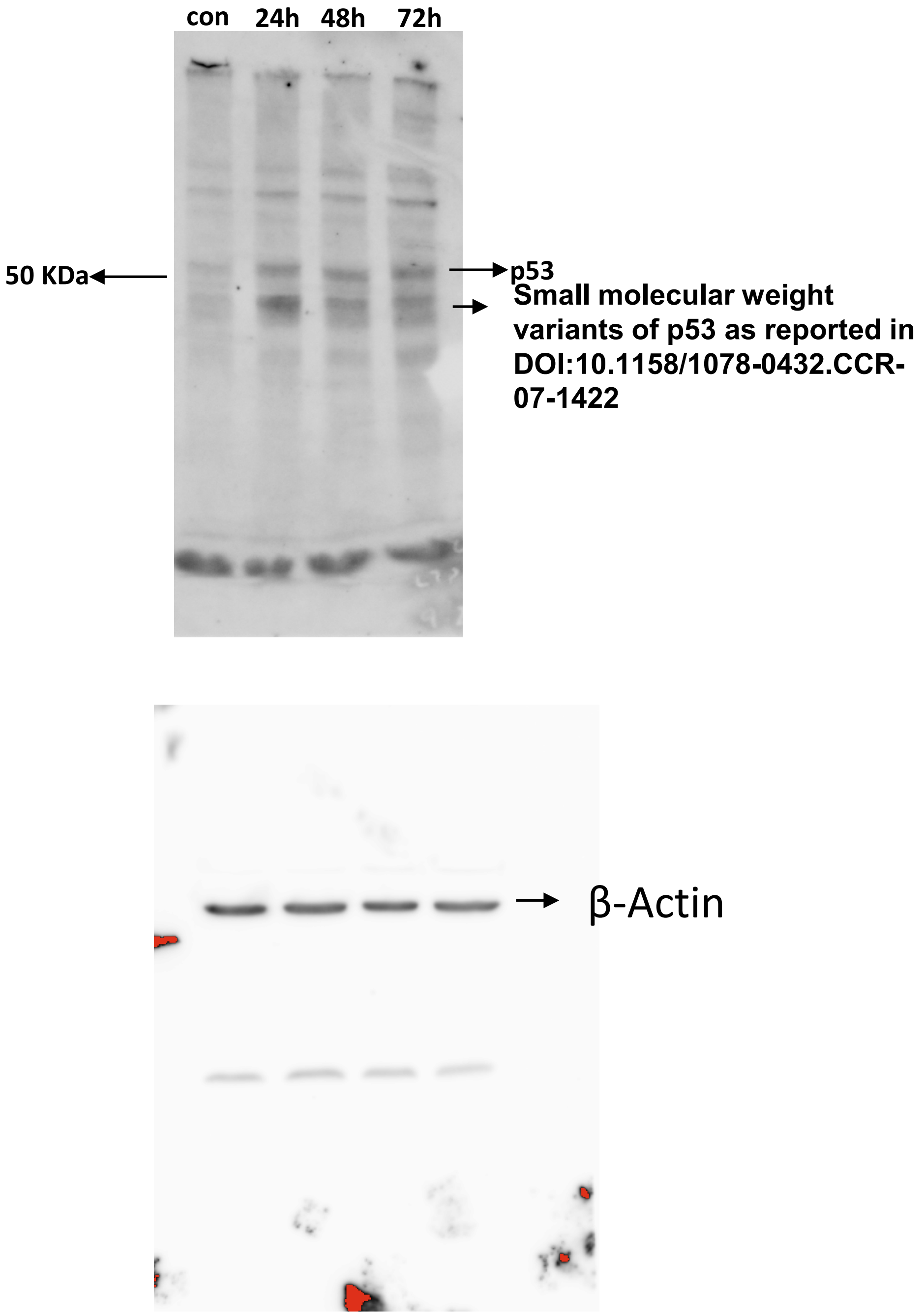

Supplementary Figure S7

Full blots of data shown in Figure 3B.
We hypothesized that DNA damage seen with atovaquone treatment might be related to activation of p53. Longer-term (24, 48 and 72h) exposure to atovaquone increased the level of total p53 in ECC-1 and OVCAR-3 cell lines. Total p53 in control and atovaquone-treated cells were monitored by western blotting. *B*-actin served as a loading control.

Supplementary Figure S8

Figure 3B (Lower Panel)

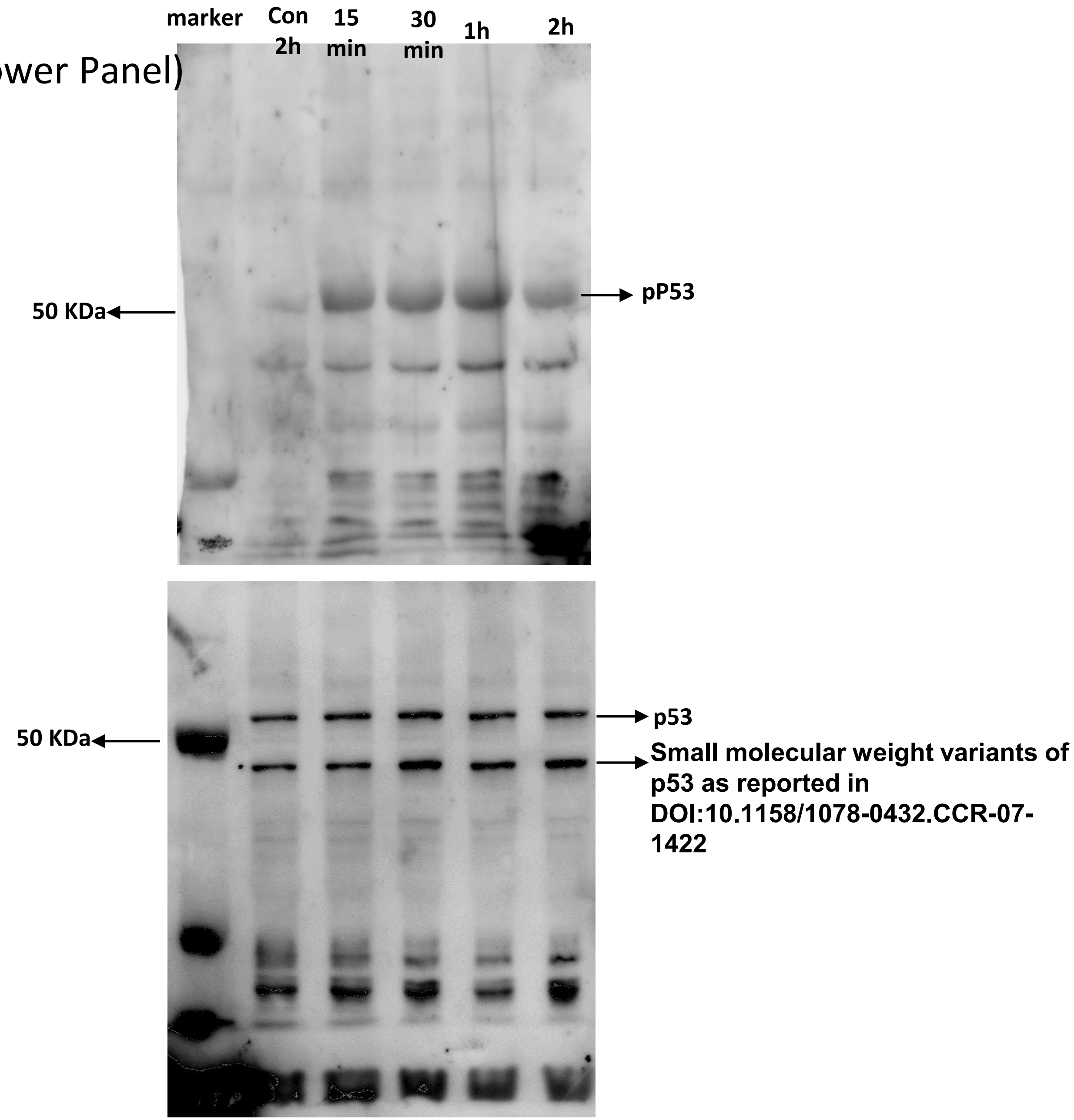

OVCAR3

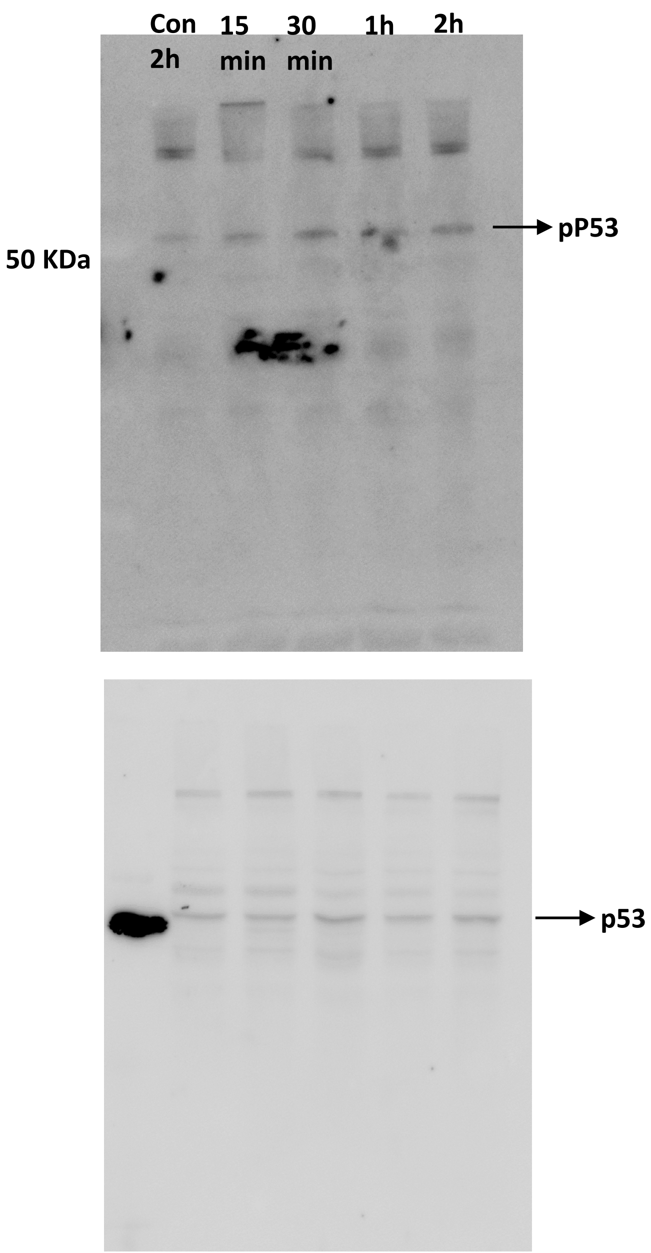

Supplementary Figure S8

Full blots of data shown in Figure 3B.

We hypothesized that DNA damage seen with atovaquone treatment might be related to activation of p53. Phosphorylated (serine-15 residue) p53 in control and atovaquone-treated cells were monitored by western blotting. B-actin served as a loading control. pP53 = phosphorylated p53.
